# *catena*-Poly[[bis­(2-amino­ethane­sulfon­ato-κ^2^ *N*,*O*)nickel(II)]-μ-1,4-bis­(1*H*-imid­azol-1-yl)benzene-κ^2^ *N* ^3^:*N* ^3′^]

**DOI:** 10.1107/S1600536811009238

**Published:** 2011-03-15

**Authors:** Jin-Biao Liu

**Affiliations:** a Chemistry and Chemical Engineering, Tianjin University of Technology, Tianjin 300384, People’s Republic of China

## Abstract

In the hydro­thermally prepared title coordination polymer, [Ni(C_2_H_6_NO_3_S)_2_(C_12_H_10_N_4_)]_*n*_, the Ni^II^ ion and the 1,4-bis­(1*H*-imidazol-1-yl)benzene ligand occupy special positions on inversion centers. The metal ion shows a slightly distorted octa­hedral coordination geometry, being linked to two N atoms of two 1,4-bis­(imidazol-1-yl)benzene ligands and to two O and two N atoms of two chelating 2-amino­ethane­sulfonate ligands. The 1,4-bis­(imidazol-1-yl)benzene ligands bridge symmetry-related Ni^II^ ions forming polymeric chains along the [110] direction.

## Related literature

For some examples of transition metal complexes of 2-amino­ethane­sulfonic acid (taurine), see: Cai *et al.* (2004 ▶, 2006 ▶); Jiang *et al.* (2006 ▶, 2005 ▶).
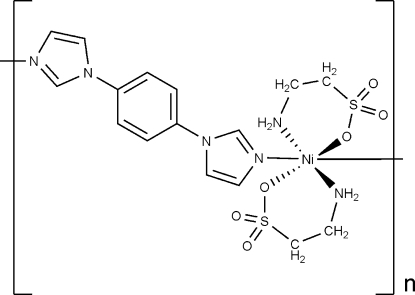


## Experimental

### 

#### Crystal data

[Ni(C_2_H_6_NO_3_S)_2_(C_12_H_10_N_4_)]
*M* *_r_* = 517.23
Monoclinic, 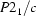

*a* = 7.4559 (15) Å
*b* = 11.494 (2) Å
*c* = 12.481 (3) Å
β = 96.19 (3)°
*V* = 1063.4 (4) Å^3^
*Z* = 2
Mo *K*α radiation
μ = 1.16 mm^−1^
*T* = 295 K
0.15 × 0.13 × 0.10 mm

#### Data collection

Bruker SMART APEX CCD diffractometer
Absorption correction: multi-scan (*SADABS*; Sheldrick, 1996 ▶) *T*_min_ = 0.841, *T*_max_ = 0.891
10967 measured reflections
2426 independent reflections
1974 reflections with *I* > 2σ(*I*)
*R*_int_ = 0.056

#### Refinement

*R*[*F* ^2^ > 2σ(*F* ^2^)] = 0.049
*wR*(*F* ^2^) = 0.097
*S* = 1.14
2426 reflections
142 parameters
H-atom parameters constrained
Δρ_max_ = 0.28 e Å^−3^
Δρ_min_ = −0.47 e Å^−3^

### 

Data collection: *SMART* (Bruker, 2007 ▶); cell refinement: *SAINT* (Bruker, 2007 ▶); data reduction: *SAINT*; program(s) used to solve structure: *SHELXS97* (Sheldrick, 2008 ▶); program(s) used to refine structure: *SHELXL97* (Sheldrick, 2008 ▶); molecular graphics: *SHELXTL* (Sheldrick, 2008 ▶); software used to prepare material for publication: *SHELXTL*.

## Supplementary Material

Crystal structure: contains datablocks global, I. DOI: 10.1107/S1600536811009238/gk2349sup1.cif

Structure factors: contains datablocks I. DOI: 10.1107/S1600536811009238/gk2349Isup2.hkl

Additional supplementary materials:  crystallographic information; 3D view; checkCIF report

## Figures and Tables

**Table 1:** Selected bond lengths (Å)

N1—Ni1	2.079 (2)
Ni1—O1	2.1070 (18)
Ni1—N2	2.126 (2)

**Fig. 1.**
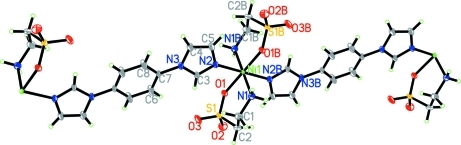
The structure of the title compound with displacement ellipsoids shown at the 30% probability level. Symmetry code for the atoms with the B label: 2-x, -y, 2-z.

**Fig. 2.**
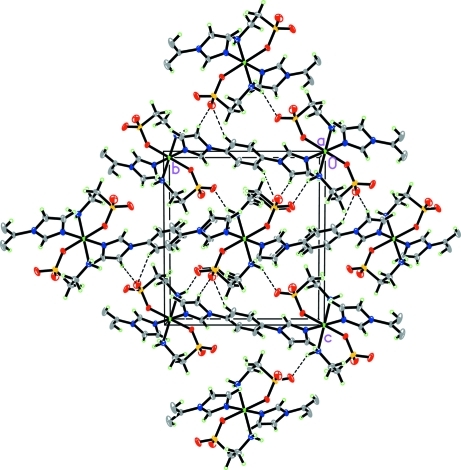
The crystal packing viewed down the a axis. Hydrogen bonds and short contacts are shown with dashed lines.

## supplementary crystallographic information

### Comment

Taurine, an amino acid containing sulfur, has important physiological functions. In fact, taurine is one of the most abundant free amino-acid-like compounds found in the heart, the skeletal muscles and the nervous system.  As part of our research on taurine complexes we report here the synthesis and crystal structure of the title nikel(II) complex with taurine and 1,4-bis(imidazol-1-yl)benzene.

The crystal structure  shows that two taurine anions chelate to the Ni^II^ ion *via* terminal N and O atoms. In addition the Ni^II^ ion is coordinated to two bridging 1,4-bis(imidazol-1-yl)benzene ligands to form one-dimensional polymer. The Ni^II ^ion and 1,4-bis(imidazol-1-yl)benzene ligand are located on inversion center. The coordination environment around nickel(II) is shown in Fig. 1. The Ni^II^ atom is six-coordinated in a distorted octahedral geometry. N-H···O and C-H···O hydrogen bonds assemble the coordination polymers into a three-dimensional supramolecular network (Fig. 2). One of the taurine N–H groups is not involved in hydrogen bonding.

### Experimental

Reagents and solvents used were of commercially available quality. A water solution (10 ml) of 2-aminoethanesulfonic acid (2.0 mmol) and KOH(2.0 mmol) was mixed with water solution (10 ml) of Ni(NO_3_)_2_.2H_2_O (1.0 mmol). 1,4-Bis(imidazol-1-yl)benzene (1 mmol) was added to the mixture, then dropped into a 23 ml Teflon-stainless steel reactor and heated at 423 K for 4 d. After cooling to room temperature, single crystals of the title compound were obtained (yield 30%). Analysis found (%): C37.27, H 4.36, N 16.32; calculated (%): C 37.14, H 4.19, N 16.24, IR (KBr, cm^-1^): 1031, 1166, 1233 (–SO_3_), 3256.6, 3300.8 (N—H).

### Refinement

All H atoms were placed in idealized positions (C—H = 0.93–0.97 Å, N—H = 0.90 Å) and refined as riding atoms with *U*_iso_(H) = 1.2*U*_eq_(C or N).

### Crystal data

**Table:**

[Ni(C_2_H_6_NO_3_S)_2_(C_12_H_10_N_4_)]	*F*(000) = 536
*M**_r_* = 517.23	*D*_x_ = 1.615 Mg m^−^^3^
Monoclinic, *P*2_1_/*c*	Mo *K*α radiation, λ = 0.71073 Å
Hall symbol: -P 2ybc	Cell parameters from 9767 reflections
*a* = 7.4559 (15) Å	θ = 3.0–27.5°
*b* = 11.494 (2) Å	µ = 1.16 mm^−^^1^
*c* = 12.481 (3) Å	*T* = 295 K
β = 96.19 (3)°	Block, blue
*V* = 1063.4 (4) Å^3^	0.15 × 0.13 × 0.10 mm
*Z* = 2	

### Data collection

**Table:**

Bruker SMART APEX CCD diffractometer	2426 independent reflections
Radiation source: fine-focus sealed tube	1974 reflections with *I* > 2σ(*I*)
graphite	*R*_int_ = 0.056
φ and ω scans	θ_max_ = 27.5°, θ_min_ = 3.3°
Absorption correction: multi-scan (*SADABS*; Sheldrick, 1996)	*h* = −9→9
*T*_min_ = 0.841, *T*_max_ = 0.891	*k* = −14→14
10967 measured reflections	*l* = −16→16

### Refinement

**Table:**

Refinement on *F*^2^	Primary atom site location: structure-invariant direct methods
Least-squares matrix: full	Secondary atom site location: difference Fourier map
*R*[*F*^2^ > 2σ(*F*^2^)] = 0.049	Hydrogen site location: inferred from neighbouring sites
*wR*(*F*^2^) = 0.097	H-atom parameters constrained
*S* = 1.14	*w* = 1/[σ^2^(*F*_o_^2^) + (0.0348*P*)^2^ + 0.7272*P*] where *P* = (*F*_o_^2^ + 2*F*_c_^2^)/3
2426 reflections	(Δ/σ)_max_ < 0.001
142 parameters	Δρ_max_ = 0.28 e Å^−^^3^
0 restraints	Δρ_min_ = −0.47 e Å^−^^3^

### Special details

**Table:**

Geometry. All e.s.d.'s (except the e.s.d. in the dihedral angle between two l.s. planes) are estimated using the full covariance matrix. The cell e.s.d.'s are taken into account individually in the estimation of e.s.d.'s in distances, angles and torsion angles; correlations between e.s.d.'s in cell parameters are only used when they are defined by crystal symmetry. An approximate (isotropic) treatment of cell e.s.d.'s is used for estimating e.s.d.'s involving l.s. planes.
Refinement. Refinement of *F*^2^ against ALL reflections. The weighted *R*-factor *wR* and goodness of fit *S* are based on *F*^2^, conventional *R*-factors *R* are based on *F*, with *F* set to zero for negative *F*^2^. The threshold expression of *F*^2^ > σ(*F*^2^) is used only for calculating *R*-factors(gt) *etc*. and is not relevant to the choice of reflections for refinement. *R*-factors based on *F*^2^ are statistically about twice as large as those based on *F*, and *R*- factors based on ALL data will be even larger.

### Fractional atomic coordinates and isotropic or equivalent isotropic displacement parameters (Å^2^)

**Table:**

	*x*	*y*	*z*	*U*_iso_*/*U*_eq_	
N1	0.8455 (3)	−0.0438 (2)	0.85704 (19)	0.0309 (6)	
H1A	0.8967	−0.1070	0.8304	0.037*	
H1B	0.7365	−0.0661	0.8744	0.037*	
C1	0.8139 (5)	0.0411 (3)	0.7680 (3)	0.0441 (9)	
H1C	0.7468	0.1069	0.7916	0.053*	
H1D	0.7420	0.0050	0.7076	0.053*	
Ni1	1.0000	0.0000	1.0000	0.01926 (15)	
S1	1.10651 (11)	0.18961 (6)	0.81832 (6)	0.0343 (2)	
N2	0.7881 (3)	0.11099 (19)	1.03850 (19)	0.0270 (5)	
O1	1.1241 (3)	0.14000 (16)	0.92774 (15)	0.0286 (5)	
N3	0.6253 (3)	0.27144 (19)	1.04500 (18)	0.0252 (5)	
C7	0.5608 (4)	0.3874 (2)	1.0207 (2)	0.0233 (6)	
O3	0.9938 (3)	0.29267 (19)	0.8110 (2)	0.0523 (7)	
O2	1.2806 (3)	0.2066 (2)	0.7786 (2)	0.0556 (7)	
C3	0.7630 (4)	0.2182 (2)	1.0020 (2)	0.0310 (7)	
H3	0.8314	0.2529	0.9527	0.037*	
C5	0.6610 (4)	0.0958 (3)	1.1103 (3)	0.0363 (8)	
H5	0.6455	0.0278	1.1486	0.044*	
C4	0.5626 (4)	0.1936 (3)	1.1170 (3)	0.0352 (8)	
H4	0.4714	0.2061	1.1609	0.042*	
C6	0.6539 (5)	0.4610 (3)	0.9619 (3)	0.0551 (11)	
H6	0.7584	0.4353	0.9349	0.066*	
C2	0.9901 (5)	0.0834 (3)	0.7320 (3)	0.0456 (9)	
H2A	0.9659	0.1163	0.6604	0.055*	
H2B	1.0690	0.0169	0.7270	0.055*	
C8	0.4054 (5)	0.4257 (3)	1.0584 (3)	0.0551 (11)	
H8	0.3393	0.3757	1.0977	0.066*	

### Atomic displacement parameters (Å^2^)

**Table:**

	*U*^11^	*U*^22^	*U*^33^	*U*^12^	*U*^13^	*U*^23^
N1	0.0350 (14)	0.0287 (13)	0.0289 (14)	−0.0018 (11)	0.0032 (11)	−0.0013 (11)
C1	0.051 (2)	0.046 (2)	0.0330 (19)	0.0049 (17)	−0.0058 (16)	0.0065 (15)
Ni1	0.0240 (3)	0.0133 (2)	0.0217 (3)	0.0050 (2)	0.00797 (19)	0.00226 (19)
S1	0.0476 (5)	0.0250 (4)	0.0340 (4)	0.0060 (3)	0.0211 (4)	0.0105 (3)
N2	0.0303 (13)	0.0198 (12)	0.0326 (14)	0.0079 (10)	0.0114 (11)	0.0040 (10)
O1	0.0359 (12)	0.0209 (10)	0.0307 (11)	−0.0019 (9)	0.0104 (9)	0.0061 (8)
N3	0.0255 (13)	0.0181 (12)	0.0339 (13)	0.0077 (10)	0.0114 (11)	0.0028 (10)
C7	0.0245 (14)	0.0172 (13)	0.0289 (15)	0.0076 (11)	0.0061 (12)	0.0001 (11)
O3	0.0687 (18)	0.0327 (13)	0.0596 (16)	0.0189 (12)	0.0253 (13)	0.0237 (11)
O2	0.0602 (17)	0.0518 (16)	0.0625 (17)	−0.0040 (12)	0.0420 (14)	0.0083 (13)
C3	0.0374 (17)	0.0217 (15)	0.0375 (17)	0.0116 (12)	0.0199 (14)	0.0063 (12)
C5	0.0379 (18)	0.0232 (16)	0.051 (2)	0.0074 (13)	0.0217 (16)	0.0092 (14)
C4	0.0314 (17)	0.0276 (16)	0.050 (2)	0.0064 (13)	0.0205 (15)	0.0082 (14)
C6	0.050 (2)	0.0357 (18)	0.088 (3)	0.0263 (17)	0.050 (2)	0.0252 (19)
C2	0.070 (3)	0.044 (2)	0.0235 (17)	0.0024 (18)	0.0096 (16)	0.0078 (14)
C8	0.052 (2)	0.0331 (19)	0.089 (3)	0.0228 (17)	0.048 (2)	0.0285 (19)

### Geometric parameters (Å, °)

**Table:**

N1—C1	1.479 (4)	N3—C3	1.355 (3)
N1—Ni1	2.079 (2)	N3—C4	1.384 (4)
N1—H1A	0.9000	N3—C7	1.438 (3)
N1—H1B	0.9000	C7—C6	1.359 (4)
C1—C2	1.513 (5)	C7—C8	1.369 (4)
C1—H1C	0.9700	C3—H3	0.9300
C1—H1D	0.9700	C5—C4	1.349 (4)
Ni1—O1	2.1070 (18)	C5—H5	0.9300
Ni1—N2	2.126 (2)	C4—H4	0.9300
S1—O3	1.450 (2)	C6—H6	0.9300
S1—O2	1.452 (2)	C2—H2A	0.9700
S1—O1	1.473 (2)	C2—H2B	0.9700
S1—C2	1.790 (4)	C8—C6^i^	1.390 (4)
N2—C3	1.320 (3)	C8—H8	0.9300
N2—C5	1.384 (4)		
			
C1—N1—Ni1	120.9 (2)	O1—S1—C2	106.43 (13)
C1—N1—H1A	107.1	C3—N2—C5	105.1 (2)
Ni1—N1—H1A	107.1	C3—N2—Ni1	124.28 (19)
C1—N1—H1B	107.1	C5—N2—Ni1	130.42 (18)
Ni1—N1—H1B	107.1	S1—O1—Ni1	133.89 (13)
H1A—N1—H1B	106.8	C3—N3—C4	106.8 (2)
N1—C1—C2	111.2 (3)	C3—N3—C7	125.9 (2)
N1—C1—H1C	109.4	C4—N3—C7	127.4 (2)
C2—C1—H1C	109.4	C6—C7—C8	119.1 (3)
N1—C1—H1D	109.4	C6—C7—N3	120.8 (2)
C2—C1—H1D	109.4	C8—C7—N3	120.1 (3)
H1C—C1—H1D	108.0	N2—C3—N3	111.7 (2)
N1^ii^—Ni1—N1	180.0	N2—C3—H3	124.1
N1^ii^—Ni1—O1^ii^	92.64 (9)	N3—C3—H3	124.1
N1—Ni1—O1^ii^	87.36 (9)	C4—C5—N2	110.5 (3)
N1^ii^—Ni1—O1	87.36 (9)	C4—C5—H5	124.8
N1—Ni1—O1	92.64 (9)	N2—C5—H5	124.8
O1^ii^—Ni1—O1	180.0	C5—C4—N3	105.9 (3)
N1^ii^—Ni1—N2^ii^	89.01 (10)	C5—C4—H4	127.0
N1—Ni1—N2^ii^	90.99 (10)	N3—C4—H4	127.0
O1^ii^—Ni1—N2^ii^	90.57 (8)	C7—C6—C8^i^	120.8 (3)
O1—Ni1—N2^ii^	89.43 (8)	C7—C6—H6	119.6
N1^ii^—Ni1—N2	90.99 (10)	C8^i^—C6—H6	119.6
N1—Ni1—N2	89.01 (10)	C1—C2—S1	114.9 (2)
O1^ii^—Ni1—N2	89.43 (8)	C1—C2—H2A	108.5
O1—Ni1—N2	90.57 (8)	S1—C2—H2A	108.5
N2^ii^—Ni1—N2	180.000 (1)	C1—C2—H2B	108.5
O3—S1—O2	113.74 (15)	S1—C2—H2B	108.5
O3—S1—O1	111.58 (13)	H2A—C2—H2B	107.5
O2—S1—O1	112.03 (14)	C7—C8—C6^i^	120.1 (3)
O3—S1—C2	106.22 (17)	C7—C8—H8	119.9
O2—S1—C2	106.24 (16)	C6^i^—C8—H8	119.9

Symmetry codes: (i) −*x*+1, −*y*+1, −*z*+2; (ii) −*x*+2, −*y*, −*z*+2.

### Hydrogen-bond geometry (Å, °)

**Table:**

*D*—H···*A*	*D*—H	H···*A*	*D*···*A*	*D*—H···*A*
N1—H1a···O3^iii^	0.90	2.33	3.148 (3)	151
C3—H3···O1	0.93	2.59	3.075 (4)	113
C3—H3···O3	0.93	2.29	3.203 (4)	165
C4—H4···O2^iv^	0.93	2.37	3.274 (4)	163
C8—H8···O2^iv^	0.93	2.53	3.359 (4)	149

Symmetry codes: (iii) −*x*+2, *y*−1/2, −*z*+3/2; (iv) *x*−1, −*y*+1/2, *z*+1/2.
